# Benzodiazepine Receptor Agonists Prescribing for Insomnia Among Adults in Primary Health Care Facilities in Beijing, China

**DOI:** 10.1001/jamanetworkopen.2023.0044

**Published:** 2023-02-17

**Authors:** Mengyuan Fu, Yuezhen Zhu, Zhiwen Gong, Can Li, Huangqianyu Li, Luwen Shi, Xiaodong Guan

**Affiliations:** 1 Department of Pharmacy Administration and Clinical Pharmacy, School of Pharmaceutical Sciences, Peking University, Beijing, China; 2 International Research Center for Medicinal Administration, Peking University, Beijing, China

## Abstract

This cross-sectional study examines the benzodiazepine receptor agonists (BZRA) prescribing rate for insomnia among adult patients in primary health care facilities (PHFs) in China.

## Introduction

Insomnia is a common sleep concern, affecting 5% to 50% of populations. It increases the risk of daytime adverse consequences and may burden patients financially.^1^ Benzodiazepine receptor agonists (BZRAs) are commonly used pharmaceutical treatments when first-line cognitive behavioral therapy for insomnia (CBT-I) fails or is unavailable.^1,2^ However, inappropriate use of BZRAs, especially among older populations, is associated with drug-related impairment.^3^ This study aimed to describe the BZRA prescribing rate for insomnia among adult patients in primary health care facilities (PHFs) in China.

## Methods

In this cross-sectional study of 67 PHFs in Dongcheng district in Beijing, outpatient visits of patients who were (1) aged 18 years and above, (2) diagnosed with insomnia, (3) had no diagnosis of anxiety or depression, and (4) prescribed with at least 1 BZRA were included. Deidentified information including patient demographics, diagnoses, and medications prescribed were digitally extracted. The primary outcome was the prescribing rate of benzodiazepines between 2016 and 2020. The secondary outcome was the mean daily dosage of each BZRA in 2020, compared with recommendations of relevant clinical guidelines in China and the US.^4,5^ Descriptive statistics were used to illustrate outcomes. Ethical approval was obtained from the Peking University institutional review board. A waiver of informed consent was granted because the data were deidentifed. The study followed the STROBE reporting guideline. Methods were detailed in the eMethods in Supplement 1. Statistical analysis was performed from March to December 2022 using Stata MP version 16.0 (StataCorp).

## Results

A total of 13 049 144 outpatient visits (7 488 425 [57.4%] by female patients) were identified between 2016 and 2020, of which 551 738 visits (321 988 [58.4%] by female patients) were eligible for inclusion. The overall prescribing rate of benzodiazepines increased by a total of 28.0 percentage points from 2016 (18 910 of 54 350 [34.8%]) to 2020 (111 271 of 177 065 [62.8%]). The largest increases were found in patients aged at least 85 years (35.2 percentage points [33.1% in 2016 to 68.3% in 2020]) and 75 to 84 years (30.3 percentage points [35.1% in 2016 to 65.4% in 2020]) (Figure). In 2020, the prescribing rate of benzodiazepines increased with patient age (18 to 44 years: 2236 of 4077 [54.8%]; 45 to 64 years: 32 496 of 54 021 [60.2%]; 65 to 74 years: 34 557 of 55 885 [61.8%]; 75 to 84 years: 25 433 of 38 859 [65.4%]; 85 years and above: 16 549 of 24 223 [68.3%]).

**Figure.**
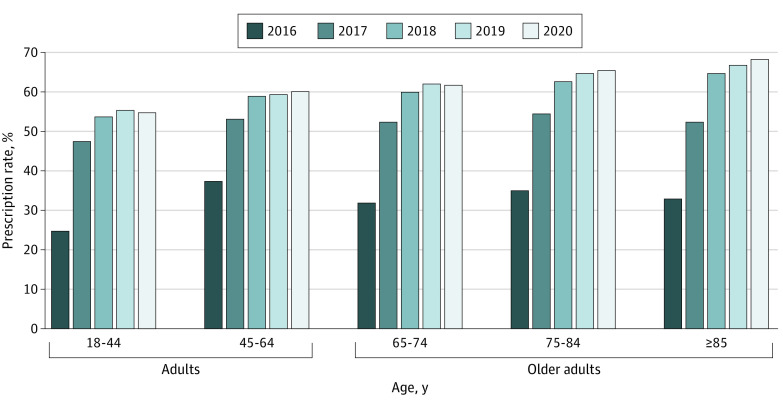
Trends in the Rate of Benzodiazepine Prescribing by Age Group in Primary Health Care Facilities in Beijing, 2016 to 2020 The figure shows the upward trend in rate of benzodiazepine prescribing among 5 age groups from 2016 to 2020.

Estazolam (109 284 of 177 883 [61.4%]) and zopiclone (65 194 of 177 883 [36.6%]) were the most commonly prescribed BZRAs in 2020. The mean daily dosages of all prescribed BZRAs for older adults were similar to those for adults. Most BZRAs were prescribed for older adults at a dosage 2 times higher than recommendations in guidelines (Table).

**Table.** Mean Daily Dosage of Different Benzodiazepine Receptor Agonists Prescribed in Dongcheng District in 2020 and Relevant Guideline Recommendations in China and the US

Medications	Frequency, No. (%)	Daily dosage, mean (SD), mg		Guideline recommendation					
		Adults	Older adults	In China^a^			In the US^b^		
				Recommend	Dosage, mg		Recommend	Dosage, mg	
					Adults	Older adults		Adults	Older adults
Benzodiazepines									
Estazolam	109 284 (61.4)	1.9 (0.4)	1.9 (0.4)	Yes	1-2	0.5	Yes	1-2	0.5
Lorazepam	1568 (0.9)	1.1 (0.4)	1.2 (0.5)	Yes	2-4	NA	NM	NA	NA
Clonazepam	176 (0.1)	3.3 (1.1)	3.5 (1.3)	NM	NA	NA	NM	NA	NA
Diazepam	137 (0.1)	4.6 (1.5)	4.6 (1.2)	Yes	5-10	NA	NM	NA	NA
Alprazolam	141 (0.1)	0.8 (0.3)	1.0 (0.3)	Yes	0.4-0.8	NA	NM	NA	NA
Nitrazepam	3 (<0.1)	30	25 (7.1)	NM	NA	NA	NM	NA	NA
Midazolam	2 (<0.1)	NA	15 (0.0)	NM	NA	NA	NM	NA	NA
Flurazepam	NA	NA	NA	Yes	15-30	15	Yes	15-30	15
Quazepam	NA	NA	NA	Yes	7.5-15	NA	Yes	7.5-15	7.5
Temazepam	NA	NA	NA	Yes	15-30	7.5-15	Yes	7.5-30	7.5
Triazolam	NA	NA	NA	Yes	0.125-0.5	NA	Yes	0.125-0.5	0.125-0.25
Z-drugs									
Zopiclone	65 194 (36.6)	7.6 (1.6)	7.6 (1.5)	Yes	7.5	3.75	NM	NA	NA
Zolpidem	1378 (0.8)	10.0 (0.9)	10.1 (1.0)	Yes	10	5	Yes	Men: 5-10; women: 5	5
Zaleplon	NA	NA	NA	Yes	5-10	5-10	Yes	10	5
Eszopiclone	NA	NA	NA	Yes	1-3	1-2	Yes	1-3	1-2

Abbreviations: NA, not applicable; NM, not mentioned; Z-drugs, nonbenzodiazepine γ-aminobutyric acid receptor agonist agents.

^a^ Guidelines for the diagnosis and treatment of insomnia in adults in China by the Chinese Medical Association (2017).^4^

^b^ Pharmacologic Treatment of Insomnia Disorder: An Evidence Report for a Clinical Practice Guideline by the American College of Physicians (2016).^5^

## Discussion

This study observed that benzodiazepines were substantially overprescribed and showed a continued upward trend of use for adult patients with insomnia in Chinese PHFs. Although guidelines and expert consensus, such as the Beers criteria, stated that treatment decisions for older adults should be more discreet and avoid routine use of benzodiazepines,^3,4^ we still observed a more prominent increase of benzodiazepine prescribing in older adults despite their vulnerability to benzodiazepine-related adverse events. Moreover, BZRAs prescribed for older adults should generally be at dosages half of those recommended for adults,^4,5^ yet, we found no difference in the prescribed dosages of BZRAs between adults and older adults treated at Chinese PHFs. Deviations from guideline recommendations and excessive use of benzodiazepines might be explained by clinicians’ limited knowledge about guideline recommendations and risks entailed in their overprescribing, insufficient clinician-patient communications, the culture of overprescribing that is also commonly observed in other health conditions in China, and that relevant Chinese guidelines are ambiguous about strategies to taper dosages of or discontinue benzodiazepines. Thus, tailored training and multidisciplinary work should be designed to promote evidence-based treatment at PHFs. Country-level campaigns to curb overprescribing of BZRAs, which managed to reduce national-level benzodiazepine use in the US,^6^ may also be promising.

Several limitations of this study should be noted. First, as our data were collected at the prescription level, we could not differentiate treatment phase (initiation or intensification) and eliminate the influence of repeated visits. Second, we could not assess and rule out diagnoses of insomnia symptoms. Third, results generated from PHFs in Dongcheng district may not be fully representative of PHFs in Beijing.

## Notes

Supplement 1. **eMethods.** Supplemental Methods

Supplement 2.
Data Sharing Statement
