# Supplementary material for: Benzodiazepine Receptor Agonists Prescribing for Insomnia Among Adults in Primary Health Care Facilities in Beijing, China
Source: JAMA Netw Open. 2023 Feb 17;6(2):e230044. doi: 10.1001/jamanetworkopen.2023.0044 (PMC9938431; doi:10.1001/jamanetworkopen.2023.0044)
Supplement: Supplement 1. — eMethods. Supplemental Methods [file jamanetwopen-e230044-s001.pdf]

Supplemental Online Content

Fu M, Zhu Y, Gong Z, et al. Benzodiazepine receptor agonists prescribing for insomnia among adults in primary health care facilities in Beijing, China. *JAMA Netw Open.* 2023;6(2):e230044.
doi:10.1001/jamanetworkopen.2023.0044

eMethods. Supplemental Methods

This supplemental material has been provided by the authors to give readers additional information about their work.

© 2023 Fu M et al. *JAMA Network Open.*

eMethods. Supplementary Methods

The ethical approval was obtained from the Peking University Institution Review Board. We followed the Strengthening the Reporting of Observational Studies in Epidemiology (STROBE) guideline.

Sampling and data collection

Settings

All 67 PHFs in Dongcheng district in Beijing were included in our analysis. All these facilities are responsible for providing basic outpatient care and public health services to individuals and families residing in the community，as the first site of contact of patients with the national healthcare system.

Visits

Outpatient visits of patients aged 18 and above and diagnosed with insomnia [International Classification of Diseases, Tenth Revision, Clinical Modification (ICD-10-CM) codes F51.0X and G47.0X],[1] and prescribed with at least one benzodiazepine receptor agonist (BZRA) between 2016 and 2020 were eligible for inclusion in our study. Visits also had diagnosis of anxiety (ICD-10-CM codes F40.X and F41.X) or depression (ICD-10-CM codes F32.X and F33.X) were excluded.[1] Detailed inclusion and exclusion process was presented in **eFigure 1**. We extracted information about date of outpatient visit, patient demographics, diagnoses, and medications prescribed from the electronic health records of the 67 PHFs. All data were digitally transferred and verified.

© 2023 Fu M et al. *JAMA Network Open.*

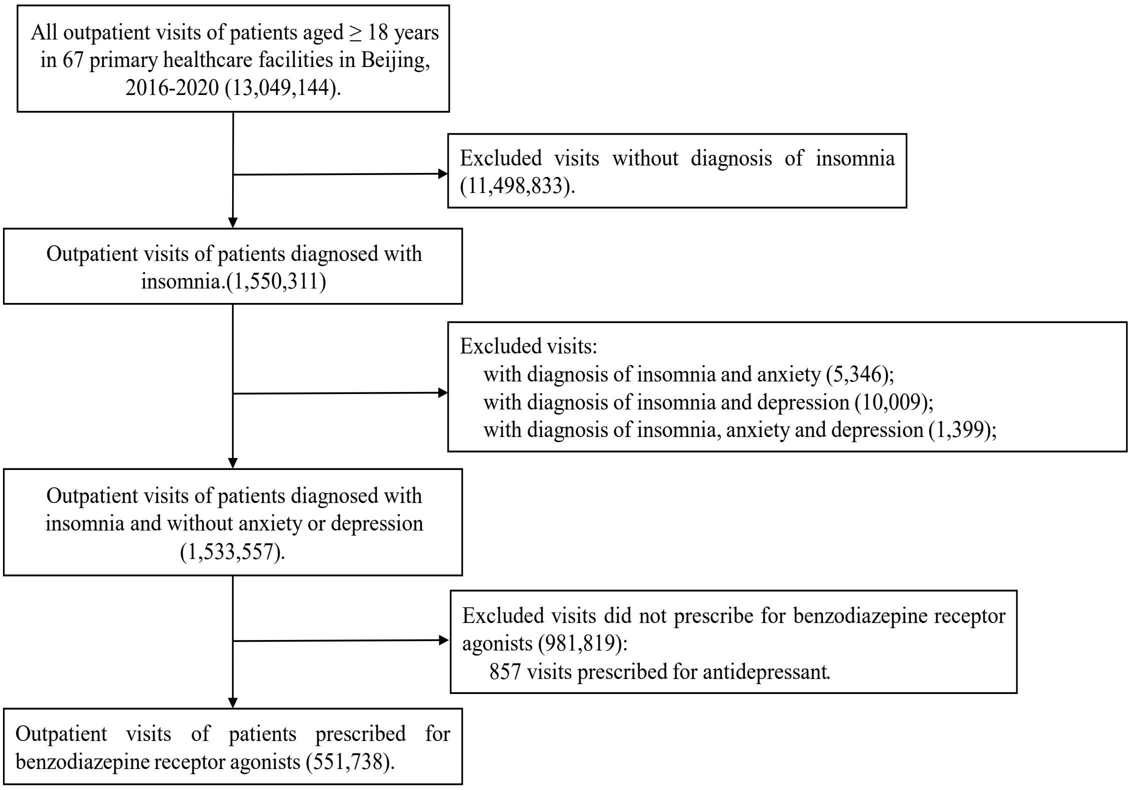

eFigure 1. Study flow diagram of inclusion and exclusion of prescriptions prescribed for benzodiazepine receptor agnoists.

Assessment of BZRAs

BZRAs included benzodiazepines and nonbenzodiazepine γ-aminobutyric acid receptor agonist agents (z-drugs). We assessed BZRAs corresponding to the Anatomical Therapeutic Chemical (ATC) classifications.[2] Benzodiazepines included N05BA (benzodiazepine derivatives of anxiolytics group), N05CD (benzodiazepine derivatives of hypnotics and sedatives group), N03AE01 (clonazepam); z-drugs were N05CF (benzodiazepine related drugs of hypnotics and sedatives group). BZRAs were listed in **eTable 1**.

eTable 1. List of benzodiazepine receptor agonists.

ATC code	Medications included
N05BA	Diazepam*; chlordiazepoxide; medazepam; oxazepam; potassium clorazepate; lorazepam*; adinazolam; bromazepam; clobazam; ketazolam; prazepam; alprazolam*; halazepam; pinazepam; camazepam; nordazepam; fludiazepam; loflazepate; etizolam; clotiazepam; cloxazolam; tofisopam; bentazepam; mexazolam; lorazepam, combinations;

© 2023 Fu M et al. *JAMA Network Open.*

N05CD	Flurazepam; nitrazepam*; flunitrazepam; estazolam*; triazolam; lormetazepam; temazepam; midazolam*; brotizolam; quazepam; loprazolam; doxefazepam; cinolazepam; remimazolam; nimetazepam
N03AE01	Clonazepam*
N05CF	Zopiclone*; zolpidem*; zaleplon; eszopiclone

- Medications marked with "*" were used in Dongcheng district in this study.

Outcome Measures

The primary outcome was the prescribing rate of benzodiazepines between 2016 and 2020, with the numerator being the number of insomnia visits prescribed with benzodiazepines and the denominator being the number of insomnia visits prescribed with BZRAs. Secondary outcome was the average daily dosage of each BZRA in 2020, compared with recommendations of relevant clinical guidelines in China and the US.[3,4] Detailed comparison of relevant treatment strategies, drug selection and drug dosages between guidelines in China and some developed countries were presented in **eTable 2** and **eTable 3**.

© 2023 Fu M et al. *JAMA Network Open.*

eTable 2. Main recommendations of insomnia treatment in the clinical guidelines of the US, EU, Canada, Korea and China.

**Guidelines**	**Recommendations for adults**		
	**Treatment strategies**	**Recommended drug/drug classes**	**Special populations**
US (ACP, 2016, part I[a] and part II[b])	■ Chronic insomnia<br>• CBT-I is the first-line treatment<br>• ACP recommends that clinicians use a shared decision-making approach to decide whether to add pharmacological therapy in adults with chronic insomnia disorder in whom CBT-I alone was unsuccessful.	• Sleep onset insomnia: zaleplon, zolpidem*, triazolam, ramelteon<br>• Sleep maintenance insomnia: doxepin<br>• Sleep onset and/or maintenance insomnia: eszopiclone, suvorexant<br>• Sleep onset insomnia and nocturnal awakenings and/or early morning awakenings: estazolam, flurazepam, quazepam<br>• Sleep onset and maintenance insomnia and nocturnal awakenings and/or early morning awakenings: temazepam	The FDA recommends lower doses of benzodiazepine and nonbenzodiazepine hypnotics in women and in older or debilitated adults.
US (AASM, 2017) [c]	■ Chronic insomnia<br>• CBT-I is the first-line treatment<br>• Medications should be considered mainly in patients who are unable to participate in CBT-I, who still have symptoms despite participation in such treatments, or, in select cases, as a temporary adjunct to CBT-I.	• Sleep onset insomnia: triazolam, zaleplon, ramelteon;<br>• Sleep maintenance insomnia: suvorexant, doxepin;<br>• Sleep onset and maintenance insomnia: eszopiclone, zolpidem, temazepam;<br>• Not suggested: trazodone, tiagabine, melatonin, diphenhydramine, tryptophan, valerian	Pharmacokinetic and pharmacodynamic properties of many medications, including BZRAs, differ among older and younger adults, necessitating lower starting dosages.
EU (2017) [d]	■ Chronic insomnia<br>• CBT-I is the first-line treatment<br>• A pharmacological intervention can be offered if CBT-I is not sufficiently effective or not available.	• Benzodiazepines: Diazepam, flunitrazepam, flurazepam, lormetazepam, nitrazepam, oxazepam, temazepam, triazolam<br>• Z-drugs: zaleplon, zolpidem, zopiclone	/
Canada (2015) [e]	■ Acute insomnia<br>• Start medication at same time as CBT-I.<br>■ Chronic insomnia	• Benzodiazepines: temazepam (flurazepam, oxazepam, triazolam are indicated for, but not recommended for primary insomnia)	• Older adults: non-pharmacological interventions are the preferred treatment

© 2023 Fu M et al. *JAMA Network Open.*

	• CBT-I is the first-line treatment<br>• Pharmacotherapy should be considered an adjunctive therapy to cognitive and behavioral therapies in the comprehensive management of insomnia (sequential treatment and select agents according to insomnia types and comorbidities): ① first line pharmacotherapy (with strongest evidence for effectiveness and efficacy); ②second line pharmacotherapy (with moderate/variable evidence for effectiveness)	• Z-drugs: zopiclone, zolpidem<br>• Others: doxepin, trazodone, melatonin, L-tryptophan, valerian	option. If a medication is to be used, the safest and best studied sleep medication for use in the elderly is doxepin.<br>• Pregnancy and post-partum: CBT-I could be the first-line treatment based on the patient's individual situation. If medication must be prescribed, lorazepam and nortriptyline are considered safe.
Korea (2020) [f]	▪ Chronic insomnia<br>• CBT-I is the first-line treatment<br>• Hypnotic medication may be prescribed only or with CBT-I if a patient has difficulty in participating in CBT-I, or if CBT-I does not show any improvement in symptoms.	• Sleep initiation insomnia: zolpidem immediate-release, eszopiclone, zaleplon, triazolam, ramelteon;<br>• Sleep maintenance disorders: doxepin, trazodone, suvorexant (melatonin prolonged-release agents are suggested for patients in 55 years or older);<br>• Sleep initiation and maintenance disorders: zolpidem controlled-release;<br>• Not suggested: melatonin immediate-release agents, doxylamine, diphenhydramine, valerian	/
China (2017) [g]	▪ Acute insomnia<br>• Medications should be prescribed as soon as possible if patients could not complete CBT-I.<br>▪ Chronic insomnia<br>• CBT-I is the first-line treatment<br>• hypnotic treatment should be supplemented with behavioral and cognitive therapies when possible (sequential treatment):	• Sleep onset insomnia: triazolam, zaleplon, ramelteon;<br>• Sleep maintenance insomnia: doxepin;<br>• Sleep onset and/or maintenance insomnia: suvorexant, eszopiclone, zopiclone, zolpidem, temazepam, flurazepam, quazepam, alprazolam, lorazepam, diazepam (melatonin prolonged-release	• Nonpharmacological treatments are the first choice for elderly patients with insomnia, especially CBT-I.<br>• Pharmacotherapy: the recommended medications include z-drugs, melatonin

© 2023 Fu M et al. *JAMA Network Open.*

	①z-drugs; ②alternate short-intermediate acting BZRAs, melatonin receptor agonists or orexin receptor antagonists if the initial agent has been unsuccessful; ③adding sedating antidepressants, especially when used in conjunction with treating comorbid depression/anxiety	agents are suggested for patients in 55 years or older)<br>• Others: amitriptyline, trazodone, mirtazapine, agomelatine	receptor agonist, orexin receptor antagonist and low-dose doxepin.

Abbreviations: CBT-I, cognitive behavioral therapy for insomnia; z-drugs, nonbenzodiazepine γ-aminobutyric acid receptor agonist agents; BZRAs, benzodiazepine receptor agonists; ACP, the American College of Physicians; AASM, the American Academy of Sleep Medicine.

[a] Management of Chronic Insomnia Disorder in Adults: A Clinical Practice Guideline from the American College of Physicians (2016).[5]

[b] Pharmacologic Treatment of Insomnia Disorder: An Evidence Report for a Clinical Practice Guideline by the American College of Physicians (2016).[4]

[c] Clinical Practice Guideline for the Pharmacologic Treatment of Chronic Insomnia in Adults: An American Academy of Sleep Medicine Clinical Practice Guideline (2017).[6]

[d] European Guideline for the Diagnosis and Treatment of Insomnia by the European Sleep Research Society (2017).[7]

[e] Assessment to Management of Adult Insomnia by the Alberta Medical Association Towards Optimized Practice (2015).[8]

[f] Korean Clinical Practice Guideline for the Diagnosis and Treatment of Insomnia in Adults by the Korean Neuropsychiatric Association (2020).[9]

[g] Guidelines for the Diagnosis and Treatment of Insomnia in Adults in China by the Chinese Medical Association (2017).[3]

* We only listed the recommendation of zolpidem for common tablets in the guideline of the US (ACP 2016, part II) and this guideline recommended zolpidem extended-release to improve sleep onset and maintenance, zolpidem sublingual (Edluar) to improve sleep onset and zolpidem sublingual (Intermezzo) to improve sleep onset after middle-of-the-night awakenings. Guidelines in the US (AASM, 2017), EU (2017), Canada (2015) and China (2017) did not mention the recommended indication of zolpidem extended-release or sublingual.

eTable 3. Differences in dosage recommendations of benzodiazepine receptor agonists in insomnia guidelines of the US, Canada, Korea and China. [a]

**Medications**	**Dosage recommendation in the US, ACP 2016- part II [b] (mg)**		**Dosage recommendation in Canada [c] (mg)**		**Dosage recommendation in Korea [d] (mg)**		**Dosage recommendation in China [e] (mg)**	
	**Adults**	**Older Adults**	**Adults**	**Older Adults**	**Adults**	**Older Adults**	**Adults**	**Older Adults**
**Benzodiazepines**								
Estazolam	1-2	0.5	/	/	/	/	1-2	0.5
Flurazepam	15-30	15	/	/	15-30	/	15-30	15

© 2023 Fu M et al. *JAMA Network Open.*

Quazepam	7.5-15	Lowest effective dose		/	/					7.5-15	Lower dose
Temazepam	7.5-30	7.5		15-30	/		/	/		15-30	7.5-15
Triazolam	0.125-0.5	0.125-0.25		/	/		0.125-0.25	/		0.125-0.5	/
Alprazolam	/	/		/	/		/	/		0.4-0.8	/
Diazepam	/	/		/	/		/	/		5-10	/
Lorazepam	/	/		/	/		/	/		2-4	/
Flunitrazepam	/	/		/	/		1	/		/	/
Brotizolam	/	/		/	/		0.25	/		/	/
Clonazepam	/	/		/	/		0.5	/		/	/
**Z-drugs**											
Eszopiclone	1-3	1-2		/	/		1-3	/		1-3	1-2
Zopiclone	/	/		3.75-7.5	3.75-5		/	/		7.5	3.75
Zaleplon	10	5		/	/		/	/		5-10	5-10
Zolpidem*	Men: 5-10<br>Women: 5	5		5-10	/		/	/		10	5

Abbreviations: z-drugs, nonbenzodiazepine γ-aminobutyric acid receptor agonist agents

[a] We only listed the guidelines with clear medication dosage recommendation for benzodiazepine receptor agonists, the clinical practice guideline for the pharmacologic treatment of chronic Insomnia in Adults by the American Academy of Sleep Medicine (2017)[6] and the European guideline for the diagnosis and treatment of insomnia by the European Sleep Research Society (2017)[7] that without dosage recommendation were not listed in this table.

[b] Pharmacologic Treatment of Insomnia Disorder: An Evidence Report for a Clinical Practice Guideline by the American College of Physicians (2016).[4]

[c] Assessment to Management of Adult Insomnia by the Alberta Medical Association Towards Optimized Practice (2015).[8]

[d] Korean Clinical Practice Guideline for the Diagnosis and Treatment of Insomnia in Adults by the Korean Neuropsychiatric Association (2020).[9]

[e] Guidelines for the Diagnosis and Treatment of Insomnia in Adults in China by the Chinese Medical Association (2017).[3]

* We only listed the recommended dosage of zolpidem for common tablets. In the guideline of the US (ACP 2016, part II), the dosage recommendations for other forms of zolpidem were 1) 6.25-12.5 mg for male adults, 6.25mg for female and older adults (extended-release); 2) 5-10 mg for male adults, 5mg for female and older adults (sublingual, Edluar); 3) 3.5 mg for male adults, 1.75mg for female and older adults (sublingual, Intermezzo). In the guideline of Korea (2020), the dosage recommendations for other forms of zolpidem were 1) 5-10 mg for adults (immediate-release); 2) 6.25-12.5 mg for adults (controlled-release). Guidelines in Canada (2015) and China (2017) did not mention the recommended dosage of zolpidem extended-release or sublingual.

© 2023 Fu M et al. *JAMA Network Open.*

Statistical Analysis

We presented results by age group (adults: 18-44 years, 45-64 years; older adults: 65-74 years, 75-84 years, and 85+ years). Descriptive statistics were used to illustrate patient characteristics with means and standard deviations (SD) reported for continuous variables and counts and proportions reported for categorical variables. Stata MP (version16.0) was used to conduct statistical analyses.

Reference

[1] World Health Organization. ICD-10 Version:2019. https://icd.who.int/browse10/2019/en. Accessed April 8, 2022.

[2] WHO Collaborating Centre for Drug Statistics Methodology. ATC/DDD Index 2022. https://www.whocc.no/atc_ddd_index/. Accessed April 8, 2022.

[3] Sleep Disorders Group of the Chinese Medical Association, Neurology Branch. Guideline for the diagnosis and treatment of insomnia in adults in China (2017 edition). Chin J Neurol. 2018,51(05):324-335. doi:10.3760/cma.j.issn.1006-7876.2018.05.002.

[4] Wilt TJ, MacDonald R, Brasure M, et al. Pharmacologic Treatment of Insomnia Disorder: An Evidence Report for a Clinical Practice Guideline by the American College of Physicians. Ann Intern Med. 2016;165(2):103-112. doi:10.7326/M15-1781

[5] Qaseem A, Kansagara D, Forciea MA, Cooke M, Denberg TD; Clinical Guidelines Committee of the American College of Physicians. Management of Chronic Insomnia Disorder in Adults: A Clinical Practice Guideline From the American College of Physicians. Ann Intern Med. 2016;165(2):125-133. doi:10.7326/M15-2175

[6] Sateia MJ, Buysse DJ, Krystal AD, Neubauer DN, Heald JL. Clinical Practice Guideline for the Pharmacologic Treatment of Chronic Insomnia in Adults: An American Academy of Sleep Medicine Clinical Practice Guideline. J Clin Sleep Med. 2017;13(2):307-349. Published 2017 Feb 15. doi:10.5664/jcsm.6470

[7] Riemann D, Baglioni C, Bassetti C, et al. European guideline for the diagnosis and treatment of insomnia. J Sleep Res. 2017;26(6):675-700. doi:10.1111/jsr.12594

[8] Toward Optimized Practice. Assessment to Management of Adult Insomnia. Alberta Medical Association; 2015. https://actt.albertadoctors.org/CPGs/Lists/CPGDocumentList/Adult-Insomnia-CPG.pdf. Accessed December 28, 2022.

[9] Choi H, Youn S, Um YH, et al. Korean Clinical Practice Guideline for the Diagnosis and Treatment of Insomnia in Adults. Psychiatry Investig. 2020;17(11):1048-1059. doi:10.30773/pi.2020.0146

© 2023 Fu M et al. *JAMA Network Open.*
