# Supplementary material for: Benzodiazepine Receptor Agonists Prescribing for Insomnia Among Adults in Primary Health Care Facilities in Beijing, China
Source: JAMA Netw Open. 2023 Feb 17;6(2):e230044. doi: 10.1001/jamanetworkopen.2023.0044 (PMC9938431; doi:10.1001/jamanetworkopen.2023.0044)
Supplement: Supplement 2. — Data Sharing Statement [file jamanetwopen-e230044-s002.pdf]

Data Sharing Statement

Fu. Benzodiazepine Receptor Agonists Prescribing for Insomnia Among Adults in Primary Health Care Facilities in Beijing, China. *JAMA Netw Open*. Published February 17, 2023. doi:10.1001/jamanetworkopen.2023.0044

Data
Data available: Yes
Data types: Deidentified participant data
How to access data: Data of this study are available upon reasonable request from corresponding authors, please contact guanxiaodong@pku.edu.cn
When available: With publication

Supporting Documents
Document types: None

Additional Information
Who can access the data: researchers whose proposed use of the data has been approved
Types of analyses: For research
Mechanisms of data availability: After aproval of a proposal and with a signed data access agreement
